# The role of tumor-associated macrophages in lung cancer

**DOI:** 10.3389/fimmu.2025.1556209

**Published:** 2025-02-26

**Authors:** Ronghao Zhu, Jing Huang, Fenhong Qian

**Affiliations:** Department of Respiratory and Critical Care Medicine, Affiliated Hospital of Jiangsu University, Zhenjiang, China

**Keywords:** tumor-associated macrophages, lung cancer, immunomodulation, immunotherapy, TAMs

## Abstract

Lung cancer remains a leading cause of cancer-related deaths worldwide, necessitating innovative treatments. Tumor-associated macrophages (TAMs) are primary immunosuppressive effectors that foster tumor proliferation, angiogenesis, metastasis, and resistance to therapy. They are broadly categorized into proinflammatory M1 and tumor-promoting M2 phenotypes, with elevated M2 infiltration correlating with poor prognosis. Strategies aimed at inhibiting TAM recruitment, depleting TAMs, or reprogramming M2 to M1 are therefore highly promising. Key signaling pathways, such as CSF-1/CSF-1R, IL-4/IL-13–STAT6, TLRs, and CD47-SIRPα, regulate TAM polarization. Additionally, macrophage-based drug delivery systems permit targeted agent transport to hypoxic regions, enhancing therapy. Preclinical studies combining TAM-targeted therapies with chemotherapy or immune checkpoint inhibitors have yielded improved responses and prolonged survival. Several clinical trials have also reported benefits in previously unresponsive patients. Future work should clarify the roles of macrophage-derived exosomes, cytokines, and additional mediators in shaping the immunosuppressive tumor microenvironment. These insights will inform the design of next-generation drug carriers and optimize combination immunotherapies within precision medicine frameworks. Elucidating TAM phenotypes and their regulatory molecules remains central to developing novel strategies that curb tumor progression and ultimately improve outcomes in lung cancer. Importantly, macrophage-based immunomodulation may offer expanded treatment avenues.

## 1 Introduction

Lung cancer remains the foremost cause of cancer-related mortality globally, responsible for 20.4% of such deaths in the U.S. in 2024, with daily fatalities nearly double those from colorectal cancer, and surpassing combined deaths from breast, prostate, and pancreatic cancers (1–3). The mortality from lung cancer is expected to greatly exceed that of gastric and breast cancers in the coming decades (4, 5). The tumor immune microenvironment, particularly macrophages differentiated into TAMs, critically influences lung cancer progression by enhancing angiogenesis and metastasis (6–11). TAMs correlate strongly with tumor size, differentiation, invasion depth, lymph node metastasis, and TNM stage in lung cancer (12), highlighting their central role in disease dynamics. Besides, in the tumor microenvironment (TME) of cancer, TAMs express characteristic surface markers such as CD68/CD163/CD204/CD206 (13), and their polarization significantly influences tumor progression and therapeutic resistance (14–18). M2-like TAMs, in particular, are associated with a pro-tumoral environment, marked by the secretion of immunosuppressive cytokines like IL-10 and TGF-β, which not only enhance tumor growth but also hinder the immune response (19–21). These macrophages also facilitate PD-L1 expression, diminishing the effectiveness of existing immunotherapies. Strategies targeting TAMs, alongside chemotherapy and PD-1/PD-L1 inhibitors, show promising therapeutic potential in preclinical models (22–24). This review discusses TAM classification, their influence on lung cancer progression, and their therapeutic implications.

## 2 Classification of TAMs

TAMs exhibit significant plasticity, adapting to various TME conditions and polarizing into two major phenotypes: the proinflammatory M1 and the tumor-promoting M2 macrophages (25–27). M1 macrophages are typically activated by inflammatory cytokines (e.g., GM-CSF, TNF-α, IFN-γ, LPS) and are characterized by their secretion of proinflammatory cytokines (e.g., IL-1α/β, IL-6, IL-12, IL-23), which enhance cytotoxic immune responses and anti-tumor immunity (28). In contrast, M2 macrophages, which predominantly promote tumor progression, are divided into subtypes based on their cytokine responses and functional roles. These subtypes include M2a, M2b, M2c, and M2d, each associated with different aspects of tumor support. M2a macrophages, driven by IL-4 and IL-13, are primarily involved in tissue repair, promoting fibrosis and immune regulation through the secretion of TGF-β and insulin-like growth factors (29). M2b macrophages, activated by immune complexes and LPS, are characterized by their anti-inflammatory profile, releasing IL-10 and IL-6, which dampen anti-tumor immunity (30). M2c macrophages, induced by IL-10, TGF-β, and glucocorticoids, play a key role in immunosuppression and extracellular matrix remodeling, contributing to the tumor’s immune escape mechanisms (31). M2d macrophages, activated by adenosine and TLRs, secrete IL-10, TGF-β, and VEGF, promoting angiogenesis and immunosuppression, which enhances tumor progression and metastasis (32, 33). The M2a/M2c macrophages facilitate the invasion of lung cancer cells and contribute to tumor progression (34). In A549 cells, the presence of M2c macrophages induced epithelial-mesenchymal transition (EMT), which was characterized by increased expression of vimentin, fibronectin, E-cadherin, NF-kB, and CCL-17 (35). Targeting TAM polarization toward the M1 phenotype, while eliminating the M2a and particularly M2c subtypes, represents a promising approach for effective anti-cancer strategies (36).

## 3 The mechanisms of TAMs in the progression of lung cancer

### 3.1 Inhibition of tumor immunity by TAMs

The organism utilizes innate and adaptive immune mechanisms to counteract tumor initiation and progression; however, tumor cells can evade these defenses through multiple strategies (37–39). In the TME, TAMs transition from an antitumor to a pro-tumor role, chiefly impeding other immune cells’ immunomodulatory functions. This inhibition occurs via several pathways: (I) M2-type TAMs, influenced by Th2 cells within the pulmonary TME, produce immunosuppressive factors like IL-10 and TGF-β. IL-10 particularly enhances PD-L1 expression in macrophages, diminishing cytotoxic T cell activity and fostering immune tolerance (40); (II) TAMs secrete C-C motif ligand 22 (CCL-22), attracting regulatory T cells (Tregs) to the TME and dampening effector T cell function, thereby cultivating an immunosuppressive environment (41); (III) Hypoxia in the TME upregulates the expression of HIF-1α in macrophages, resulting in decreased CD8^+^ T-cell activation mediated by macrophages and promoting immune evasion (42–44). While under hypoxic conditions, TAMs produce increased levels of arginase I, which depletes L-arginine in the microenvironment, thereby inhibiting T cells by arresting them in the G0/G1 phase of the cell cycle and preventing their proliferation (45); (IV) CD206 mannose receptors on the surface of TAMs suppress CD45 phosphatase activity, leading to upregulated expression of cytotoxic T-lymphocyte–associated protein 4 (CTLA-4) and ultimately inducing T-cell tolerance (46); (V) IL-4 stimulation activates the PI3Kγ–mTor–S6Ka–C/EBPβ pathway in macrophages and inhibits nuclear factor kappa-B (NF-κB), thereby suppressing immunity and promoting tumor growth (47). In addition, TAMs interact with various immune cells, including T lymphocytes, NK cells, dendritic cells, neutrophils, and MDSCs. Moreover, TAMs suppress the cytotoxic activity of T cells, natural killer T (NKT) cells, and NK cells by expressing ligands for immune checkpoint receptors such as PD-1 and CTLA-4 (48–51).

### 3.2 TAMs promote angiogenesis in lung cancer

TAMs secrete proangiogenic factors (e.g., VEGF, CXCL8) into avascular regions, promoting tumor angiogenesis. VEGF strongly stimulates endothelial cell proliferation, neovascularization, and vascular permeability, facilitating tumor cell extravasation (52). TAM-derived IL-8, VEGF, and urokinase-type plasminogen activator (uPA) further contribute to neovascularization (53). Hypoxia in the TME induces HIF-1/2, which upregulate VEGF, platelet-derived growth factor (PDGF), and EGF (54, 55). VEGF also attracts more TAMs, generating a positive feedback loop (56). Additionally, TAMs secrete TGF-β, TNF-α, MMPs, and TIE2, all of which promote intratumoral vascular formation (57–60).

### 3.3 TAMs involved in lung cancer cell proliferation, invasion, and metastasis

In lung cancer, TAMs foster tumor proliferation, invasion, and metastasis via chemokines and cytokines—TGF-β, IL-10, IL-6, matrix metalloproteinases (MMPs), and VEGF—and may activate cancer stem cells (CSCs) through IL-10 (61). Hypoxia from rapid tumor growth increases M2 macrophages, elevating IL-10, VEGF, and HIF-1α, promoting metastasis and further macrophage infiltration (62). TAMs also facilitate EMT, critical for metastasis (63) by upregulating CRYAB (64) and Ezrin phosphorylation–mediated FUT4/LeY fucosylation (65). TAM-derived IL-6, IL-10, and TGF-β regulate EMT (66) with IL-6 and IL-10 inducing M2 polarization through JAK/STAT3 (67), and TGF-β promoting SOX9 expression via c-jun/SMAD3, enhancing proliferation and invasiveness (68, 69). Furthermore, TAMs secrete MMPs, mainly MMP-9 and MMP-2, to degrade the extracellular matrix and facilitate invasion and metastasis (70). MMP-9 expression is also associated with lymph node metastasis and prognosis (71). Chemokines like CCL18 from TAMs fuel tumor progression (72). In addition, TAMs promote collagen fiber formation, guiding lung cancer cells toward blood vessels and into circulation (73). CSCs promote lung cancer progression as well (61). TAMs interact with CSCs, including TAM recruitment through vascularization, the release of chemokines by TAMs to maintain CSC dormancy, and antigen modification of CSCs to evade immune recognition, which plays a crucial role in tumor progression and metastasis. Besides, CSCs promote TAM polarization from M1 to M2, induce angiogenesis via VEGF, and create supportive niches through tissue repair pathways (74). A potential therapeutic strategy targeting the interactions between TAMs and CSCs could provide an effective approach for lung cancer (75, 76). Exosomes also participate in tumorigenesis and metastasis by mediating material exchange among tumor cells and macrophages (77–79). For instance, exosomes derived from tumors release miR-19b-3p, which promotes the polarization of M2 macrophages, and also secrete LINC00273, facilitating the metastasis of lung adenocarcinoma through the Hippo signaling pathway (80). MiR-501-3p expression was elevated, while WDR82 levels were reduced in lung cancer tissues and cell lines. Additionally, M2-derived exosomes contributed to the further increase in miR-501-3p levels. These exosomes, along with their cargo of miR-501-3p, played a significant role in promoting lung cancer cell proliferation. Exosomal miR-501-3p has been shown to inhibit apoptotic processes, thus further enhancing lung cancer proliferation, invasion, and metastasis (81). Besides, exosomes secreted by M2 TAMs contribute to osimertinib resistance in non-small cell lung cancer by modulating the MSTRG.292666.16-miR-6836-5p-MAPK8IP3 signaling pathway (82).

### 3.4 TAMs promote drug resistance in lung cancer cells

TAMs facilitate tumor growth, progression, and chemoresistance by supplying cytokines and upregulating anti-apoptotic genes. Treatment of murine models with CTX, PTX, or DOX increases CD206^+^ TAMs, triggering revascularization and recurrence (83). Chemotherapy-induced IL-34 augments TAM-mediated drug resistance (19). Furthermore, TAM-derived extracellular matrix remodels tumor–macrophage interactions, enhancing chemoradiotherapy resistance (84, 85). M2-polarized TAMs secrete growth factors and suppress cell death signaling, conferring chemoresistance and radioprotection, ultimately leading to poor prognosis (86). Cisplatin-resistant lung cancer cells exhibit enhanced self-renewal and metastatic capacity by secreting macrophage inhibitory factor (MIF), which promotes the M2 polarization of TAMs (87). Near-infrared fluorescence (NIRF) imaging and single-photon emission computed tomography (SPECT) imaging reveal M2 TAM infiltration in recurrent tumors and lymph node metastases, underscoring their role in tumor recurrence and pulmonary spread post-chemotherapy (88). P2X7 activation of STAT6/IRF4 drives M2 polarization, fueling tumor proliferation, angiogenesis, and T-cell suppression. Inhibiting or deleting P2X7 weakens M2 TAMs, restricting lung tumor growth while overcoming immunotherapy and chemotherapy resistance (89). Besides, TAMs activated CSC-related pathways might enhance drug resistance (90).

## 4 Major molecules regulating TAM function in lung cancer

### 4.1 CSF-1/CSF-1R axis

GM-CSF governs hematopoietic cell generation and differentiation, as well as angiogenesis. CSF-1 binds CSF-1R, triggering the PI3K signaling cascade and further activating protein kinase B (AKT) and mammalian target of rapamycin complex 2 (mTORC2), thereby regulating the M1/M2 polarization axis of macrophages (91). PI3K and AKT overexpression suppresses M1 activation partly by negatively regulating the NF-κB signaling pathway, which otherwise promotes M1 phenotypes (92). CSF-1R also binds IL-34; elevated IL-34 and CSF-1R levels correlate with tumor progression and poorer survival (93). Moreover, CSF-1 can recruit and reprogram TAMs to secrete factors that facilitate tumor growth and metastasis (94).

### 4.2 IL-4/IL-13 and JAK-STAT6 signaling pathway

Inflammatory factors are pivotal in inflammatory diseases progression and significantly influence the efficacy of therapies (95–98). IL-4 and IL-13 participate in Th2-type immune responses (99) and drive TAMs toward an M2 phenotype that promotes aberrant angiogenesis and tumor progression. Both cytokines bind type I or II IL-4 receptors, activating JAKs and subsequently phosphorylating STAT6, which forms dimers, translocates to the nucleus, and upregulates M2-associated genes such as Arg-1, mannose receptor c-type 1 (Mrc-1), Chil3/Ym1, and resistin-like molecule alpha (RELM-α or Fizz-1) (100, 101). STAT6 function is also regulated by additional factors: one study found that TRAF3 enhances STAT6 ubiquitination (K450), thereby boosting transcriptional activity and M2 marker expression (102). In a B16 melanoma model, TRAF3 deficiency in bone marrow suppressed tumor growth and metastasis, underscoring STAT6’s importance in macrophage-mediated immunosuppression.

### 4.3 Toll-like receptors

TLR are crucial in recognizing pathogen-associated molecular patterns (PAMPs) and damage-associated molecular patterns (DAMPs), playing a significant role in activating the immune response (103). In the context of cancer, TLR signaling can influence TAM polarization, promoting tumor progression (104). TLRs, particularly TLR2, TLR4, and TLR7, have been shown to modulate the immune microenvironment by skewing macrophages toward a pro-tumoral M2 phenotype, thus enhancing immune evasion, angiogenesis, and metastasis (105, 106). Another study showed that combining low-toxicity IFN (IFN-γ, IFN-β) with TLR agonists increased M1 induction by up to 100-fold, suggesting novel TLR-targeted strategies for tumor immunotherapy (107). For instance, clinical trials indicated that the intravenous administration of TLR agonists poly(I:C) + R848 reduced the growth of orthotopic lung tumors through demonstrated reprogramming of interstitial macrophages increasing the M1/M2 ratio (104).

### 4.4 CD47-SIRPα

**Figure 1:**
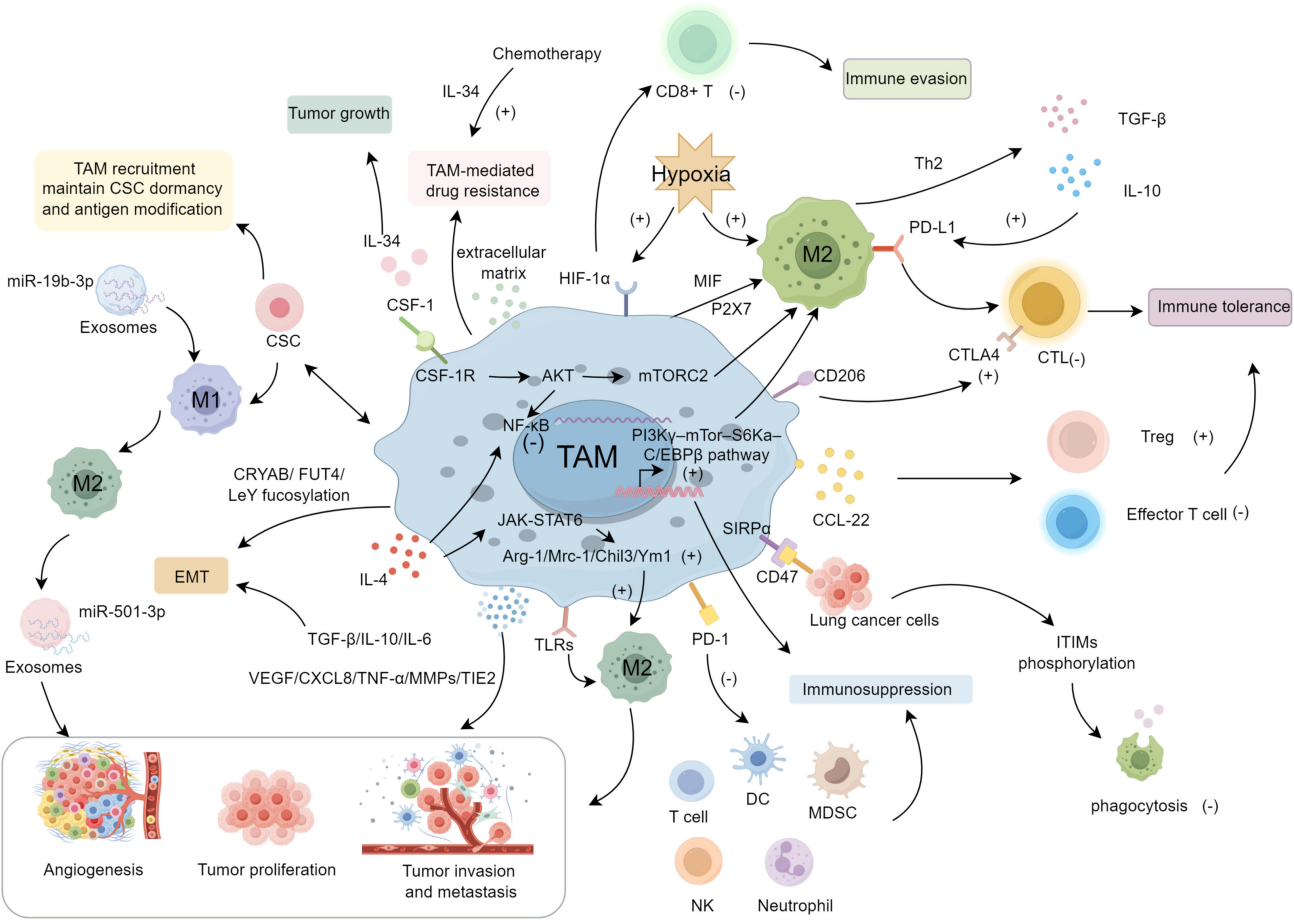
The mechanisms and regulating molecules of TAMs in the progression of lung cancer.

CD47 (integrin-associated protein) is a thrombospondin-family receptor that modulates platelet activation, migration, adhesion, and phagocytosis. Overexpressed on tumor cells, CD47 binds SIRPα on macrophages, leading to phosphorylation of immunoreceptor tyrosine-based inhibitory motifs (ITIMs) and inhibiting phagocytosis (108, 109). High CD47 expression is associated with worse prognoses in various solid tumors. In non-small cell lung cancer (NSCLC), approximately two-thirds of tumor samples exhibit CD47 overexpression, correlating with increased SIRPα on TAMs (110). Although CD68^+^ TAMs typically suggest better outcomes, high SIRPα levels on TAMs reduce FOXP3^+^ TIL proportions and TIL scores, leading to poor prognosis. In 191 resected NSCLC specimens, CD47 was more frequently overexpressed in females, never-smokers, and lung adenocarcinoma patients, significantly correlating with EGFR mutations but inversely with PD-L1 levels and tumor mutation burden (111). Gefitinib-resistant lung cancer cells upregulate CD47, impairing macrophage phagocytosis. Inhibition of CD47-SIRPα signaling via STAT3 inhibitors enhances TAM phagocytic activity and mitigates EGFR-TKI resistance, offering a novel treatment strategy for patients with acquired EGFR-TKI resistance (112) (**Figure 1**).

## 5 TAMs as therapeutic targets in lung cancer

TAMs play a critical role in the formation of the TME, tumor immunity, and responses to immunotherapy, making them important and promising therapeutic targets in lung cancer. Notably, combining therapies targeting TAMs with other immunotherapeutic approaches can yield superior efficacy (113, 114). Below, we introduce four strategies for targeting TAMs.

### 5.1 Inhibiting TAMs recruitment

Limiting TAM infiltration requires restricting the recruitment of circulating monocytes via factors like chemokines (e.g., CCL2), cytokines (e.g., CSF-1), and complement mediators. In particular, the CCL2–CCR2 and CSF-1/CSF-1R axes have garnered significant attention. CCL2–CCR2 signaling recruits TAMs, promoting tumor angiogenesis and growth (115). In a Lewis lung cancer model, CCR2 knockout or the CCR2 inhibitor RS504393 reduced TAM recruitment, shifted TAMs to an M1 phenotype, and suppressed angiogenesis and tumor progression (22). Another approach to prevent TAM infiltration into the tumor microenvironment involves inhibiting the CSF-1–CSF-1R axis, which is vital for TAM differentiation, survival, and recruitment (6). CSF-1R blockade can reduce the infiltration of TAMs mediated by immunosuppressive T cells (116). In animal models, it has been demonstrated that small-molecule CSF-1R inhibitors in combination with immune checkpoint inhibitors are currently in Phase I/II clinical trials for the treatment of advanced breast cancer and other solid tumors (117). This finding provides a novel perspective for targeting and inhibiting TAM recruitment in lung cancer.

### 5.2 Depleting TAMs

High TAM infiltration in lung cancer correlates with poor prognosis, though single-agent depletion strategies have shown limited efficacy (118–120). Specifically targeting M2-like TAMs is preferable, as broad macrophage eradication is suboptimal. For instance, selectively depleting M2 TAMs improved survival in tumor-bearing mice (121). The CSF-1/CSF-1R axis remains the most explored depletion method. Blocking CSF-1R depletes macrophages in both normal and tumor tissues, as seen with BLZ945 and PLX3397 (122). Moreover, combining CSF-1R inhibition with other treatments yields better outcomes, enhancing CD8^+^ T-cell infiltration and slowing tumor progression, especially when coupled with anti-PD-1 therapy (114, 123). Targeting surface molecules like CD52, scavenger receptor A, folate receptor β, and CD206 can also deplete TAMs (124, 125).

### 5.3 Reprogramming TAMs in lung cancer

Elevated M2 TAM density correlates with poorer survival, whereas higher M1 density predicts better outcomes (126, 127). Reprogramming M2 TAMs to M1 slows tumor progression (128). TLR7/8 agonist R848, delivered via β-cyclodextrin nanoparticles, can repolarize M2 TAMs, while combining R848 with anti-PD-1 further enhances therapeutic efficacy (129). Besides depleting macrophages, CSF-1/CSF-1R inhibitors can modulate TAM phenotypes (130, 131). Ginsenoside Rh2 also shifts M2 to M1 macrophages, reducing NSCLC metastasis by downregulating VEGF, MMP2, and MMP9 (132). Nanoformulations that load macrophage-targeted agents (e.g., TLR7/8 agonists) can overcome delivery challenges in inaccessible tumors (133, 134). Inhibiting TAM receptors, namely Tyro3, Axl, and MERTK, further counters M2-like polarization (135). Axl and MERTK are overexpressed in lung cancer (136, 137), and MERTK inhibitors (e.g., UNC2025) reduce distant metastases in NSCLC models (138). Additionally, imatinib suppresses M2 polarization by inhibiting STAT6 phosphorylation, preventing metastasis (139, 140). Blocking M2-activating cytokines (IL-13, IL-4, IL-10) also enhances efficacy, including immune checkpoint inhibitors (84), as high IL-10 in TAMs is linked to NSCLC staging (141). Cancer cells release succinate into the extracellular space, promoting macrophage migration and influencing TAM polarization. This suggests that succinate functions as an oncometabolite, potentially serving as a critical target for cancer chemoprevention and therapeutic strategies (142).

### 5.4 Macrophages as a drug delivery system

Although biocompatible nanomaterials have improved drug delivery, their limited circulation half-life remains a hurdle. Macrophages, with longer half-lives, immune functions, and intrinsic tumor-homing capabilities, offer a promising alternative (143–145). In animal models, doxorubicin-loaded M1 macrophages prolonged survival and inhibited tumor invasion (146). However, directly loading macrophages with anti-cancer drugs may impair their function. To circumvent this, “indirect” loading onto biocompatible nanomaterials allows higher drug capacity while preserving macrophage viability (147, 148). Attaching microparticles to macrophage surfaces, rather than internalization, can further maintain targeting efficiency (149). Photothermal therapy, which uses light to generate cytotoxic heat, is another emerging strategy. Nanomaterials with high photothermal conversion efficiency can be immobilized on macrophage membranes to enhance biocompatibility, immune evasion, and tumor-homing (150). For instance, doxorubicin-loaded graphene oxide on mouse macrophages markedly suppresses tumor growth (151) (**Table 1**).

**Table 1:** Therapeutic strategies targeting TAMs in lung cancer.

Strategy	Methods	Examples	Effects	References
Inhibiting TAMs Recruitment	- Targeting chemokines and their receptors (e.g., CCL2–CCR2 axis) - Inhibiting cytokines (e.g., CSF-1/CSF-1R axis) - Blocking complement mediators	- CCR2 inhibitor RS504393 - CSF-1R inhibitors	- Reduced TAM recruitment - Shifted TAMs to M1 phenotype - Suppressed angiogenesis and tumor progression	(10, 77–79)
Depleting TAMs	- Inhibiting CSF-1/CSF-1R signaling - Targeting surface molecules (e.g., CD52, scavenger receptor A, folate receptor β, CD206)	- CSF-1R inhibitors BLZ945 and PLX3397 - Anti-CD52 antibodies	- Depletion of TAMs in tumor and normal tissues - Enhanced CD8+ T-cell infiltration - Slowed tumor progression - Synergistic effects with anti-PD-1 therapy	(4, 75–85)
Reprogramming TAMs	- Repolarizing M2 to M1 phenotype using agonists (e.g., TLR7/8 agonist R848) - inhibitors (e.g., CSF-1/CSF-1R inhibitors) - Modulating TAM receptors (Tyro3, Axl, MERTK) - Blocking M2-activating cytokines	- R848-loaded β-cyclodextrin nanoparticles - Ginsenoside Rh2 - MERTK inhibitor UNC2025 - Imatinib - STAT6 inhibitors	- Shift from M2 to M1 phenotype - Reduced tumor progression and metastasis - Enhanced therapeutic efficacy - Inhibited M2-related signaling pathways	(53, 86–99)
Macrophages as a Drug Delivery System	- Direct loading of drugs onto macrophages - Indirect loading via biocompatible nanomaterials - Attaching microparticles to macrophage surfaces - Utilizing photothermal therapy with nanomaterials	- Doxorubicin-loaded M1 macrophages - Nanoparticle-conjugated TLR7/8 agonists - Doxorubicin-loaded graphene oxide on macrophages	- Targeted drug delivery to hypoxic tumor regions - Prolonged macrophage viability and function - Enhanced tumor suppression - Improved biocompatibility and immune evasion	(100–108)

## 6 Conclusion

Lung cancer remains a leading global malignancy, with persistently low five-year survival despite immunotherapy advances. Increasing evidence identifies TAMs as key immunosuppressive TME mediators driving tumor cell proliferation, angiogenesis, metastasis, immune evasion, and drug resistance. Their polarization state and infiltration levels strongly correlate with prognosis; hence, inhibiting TAM aggregation, depleting TAMs, or reprogramming M2 to M1 is critical for improving outcomes. Clinical trials show that combining TAM-targeting agents with existing therapies enhances efficacy, benefiting previously unresponsive patients. Further research is needed to elucidate macrophage-derived cytokines, exosomes, and other factors within the TME, potentially expanding therapeutic targets and advancing macrophage-based drug delivery. Clarifying TAM phenotypes and their molecular pathways is essential for understanding lung cancer progression, informing novel immunotherapeutic strategies, and providing key theoretical underpinnings for developing next-generation antitumor drugs.
